# Serum enterolactone concentrations are low in colon but not in rectal cancer patients

**DOI:** 10.1038/s41598-019-47622-6

**Published:** 2019-08-01

**Authors:** Anne Tuomisto, Natalja P. Nørskov, Päivi Sirniö, Juha P. Väyrynen, Shivaprakash J. Mutt, Kai Klintrup, Jyrki Mäkelä, Knud Erik Bach Knudsen, Markus J. Mäkinen, Karl-Heinz Herzig

**Affiliations:** 1 Cancer and Translational Medicine Research Unit, Department of Pathology, University of Oulu, Oulu, Finland; 2 Oulu University Hospital and Medical Research Center Oulu, Oulu, Finland; 3 Aarhus University, Department of Animal Science, AU-Foulum, Blichers Alle 20, P.O. Box 50, DK-8830 Tjele, Denmark; 4 Department of Oncologic Pathology, Dana-Farber Cancer Institute and Harvard Medical School, Boston, MA USA; 5 Research Unit of Biomedicine and Biocenter of Oulu, University of Oulu, Oulu, Finland; 6 Research Unit of Surgery, Anesthesia and Intensive Care, University of Oulu, Oulu, Finland; Department of Surgery, Oulu University Hospital and Medical Research Center Oulu, Oulu, Finland; 7 Department of Gastroenterology and Metabolism, Poznan University of Medical Sciences, Poznan, Poland

**Keywords:** Prognostic markers, Tumour biomarkers

## Abstract

The dietary lignan metabolite, enterolactone, has been suggested to have anti-cancer functions, and high serum enterolactone concentrations have been associated with decreased risk of breast and prostate cancers. We hypothesized that serum enterolactone concentrations as a marker of plant-based foods are associated with decreased risk in colorectal cancer (CRC). We measured serum enterolactone glucuronide and sulfate concentrations by liquid chromatography-tandem mass spectrometry in 115 CRC patients and 76 sex- and age-matched controls and analyzed the results with respect to tumor parameters, clinical parameters, and systemic inflammatory markers. Patients with colon cancer had significant lower serum enterolactone glucuronide and sulfate concentrations than controls (glucuronide: median 3.14 nM vs. 6.32 nM, P < 0.001; sulfate: median 0.13 nM vs. 0.17 nM, P = 0.002), whereas rectal cancer patients had similar enterolactone levels as controls (glucuronide: median 5.39 nM vs. 6.32 nM, P = 0.357; sulfate: median 0.19 nM vs. 0.17 nM, P = 0.452). High serum enterolactone concentrations were associated with low tumor grade, high serum creatinine levels, and concomitant diabetes. In summary, our results suggest that serum enterolactone concentrations are decreased in colon but not in rectal cancer. Further investigations are required to assess whether this reflects an altered lignan metabolism by the colon microbiome.

## Introduction

Western diets with low fiber consumption are a risk factor for colorectal cancer (CRC)^1^. High-fiber, plant-based foods, such as whole grains, seeds, some vegetables and fruits, are a rich source of phytoestrogens including flavonoids and plant lignans^2,3^. In the colon, plant lignans are metabolized by multistep processes, catalyzed by intestinal bacteria including *Moryella*, *Streptobacillus*, *Fastidiosipila* and *Acetanaerobacterium*, resulting in the production of enterolignans, enterodiol and enterolactone.

Enterolactone has a similar structure to 17B-estradiol and is capable of binding to estrogen receptors^4,5^. This may explain the reported association between enterolactone and decreased risk of hormone-sensitive cancers^6^. Indeed, enterolactone has been reported to harbor anti-tumor activities^7–9^, including inhibition of tumor growth and angiogenesis, and stimulation of apoptosis^10,11^. Systemically, enterolignans are able to modulate the immune response by suppressing lymphocyte proliferation and cytokine production^12^. The cancer-protective effects of enterolactone may be facilitated by estrogen-receptor dependent and/or independent mechanisms^7–9^.

A recent meta-analysis of 16 studies found an inverse association between lignan intake and CRC risk, but no association between circulating enterolactone and CRC risk^13^. Moreover, no correlation was observed in the EPIC-Norfolk study between phytoestrogen exposure and the risk of colorectal cancer risk in an European population^14^, whereas another study from the same population found an association between enterolactone and CRC risk among women based on a comprehensive phytoestrogen nutrient intake database^15^. In addition to sex differences, tumor localization-specific differences have been reported in the association between enterolactone and CRC risk^16,17^. However, despite its potential tumor-inhibiting effects, the alterations in preoperative serum enterolactone levels in CRC patients and their clinical significance have not been well-defined.

In this study, we analyzed serum enterolactone levels in 115 CRC patients in relation to the levels in 76 healthy controls, as well as tumor and patient characteristics, including systemic inflammatory markers.

## Results

### Serum enterolactone concentrations in CRC patients and healthy controls

Enterolactone glucuronide was the main circulating form in both CRC patients (96% of all enterolactone) and their age- and gender-matched controls (95% of all enterolactone), while sulfated enterolactone was the minor conjugation form (Table 1). The patients had significantly lower serum enterolactone glucuronide levels compared to controls (median 3.41 nM vs. 6.31 nM, respectively; P = 0.001) and showed a tendency toward lower enterolactone sulfate levels relative to controls (median 3.41 nM vs. 6.31 nM, p = 0.053). A strong correlation existed between enterolactone glucuronide and sulfate concentrations both in CRC patients (r = 0.877, P < 0.001) and in control subjects (r = 0.796, P < 0.001).

**Table 1:** Characteristics of the CRC patients and controls.

	CRC patients (n = 115)	Healthy controls (n = 76)
Age, mean (SD)	67.63 (11.26)	67.07 (10.42)
**Gender**		
Male	57 (49.6%)	38 (50.0%)
Female	58 (50.4%)	38 (50.0%)
**Tumor location**		
Proximal colon	49 (42.6%)	
Distal colon	28 (24.3%)	
Rectum	38 (33.0%)	
**WHO grade**		
Grade 1	14 (12.3%)	
Grade 2	86 (74.8%)	
Grade 3	14 (12.3%)	
**TNM stage**		
Stage I	17 (14.9%)	
Stage II	46 (40.4%)	
Stage III	30 (26.3%)	
Stage IV	21 (18.3%)	
Body mass index (BMI), median (IQR)^A^	26.4 (23.3–28.8)	
Body mass index (BMI) in patients and controls aged >65, median (IQR)^B^	26.3 (23.3–28.2)	27.2 (24.5–30.3)
Enterolactone glucuronide (nM), median (IQR)^C^	3.410 (1.01–7.31)	6.310 (2.91–10.71)
Enterolactone sulfate (nM), median (IQR)^D^	0.148 (0.062–0.254)	0.168 (0.109–0.348)

^A^Data not available for controls aged less than 65 years. ^B^CRC patients vs. healthy controls; *P* = 0.078. ^C^*P* = 0.001. ^D^*P* = 0.053. The *P* value are for Mann-Whitney test. Abbreviations: CRC: colorectal cancer; IQR, interquartile range; SD: standard deviation.

### Relationships between serum enterolactone concentrations and clinicopathological parameters

Next, we analyzed the relationships between serum enterolactone concentrations and tumor and patient characteristics (Table 2). Low serum abundance of both the glucuronide and sulfate conjugated forms were associated with tumor localization, namely colon compared to the rectum (P = 0.049 and P = 0.012, respectively). Indeed, patients with rectal cancer (n = 38) had similar enterolactone concentrations to controls (glucuronide: median 5.39 nM vs. 6.32 nM, P = 0.357; sulfate: median 0.19 nM vs. 0.17 nM, P = 0.452, Table 2), whereas the patients with colon cancer (n = 77) had significantly lower concentrations of both enterolactone forms compared with control subjects (glucuronide: median 2.61 nM vs. 6.32 nM, P < 0.001; sulfate: median 0.13 nM vs. 0.17 nM, P = 0.002, Table 2). In addition, tumor differentiation was associated with serum enterolactone levels: the CRC patients with low-grade tumors had higher enterolactone levels than the patients with poorly differentiated tumors (glucuronide: P = 0.032, sulfate: P = 0.011, Table 2). Serum enterolactone levels were not associated with patient age, gender, or body mass index (BMI), tumor stage, tumor necrosis, or the the fraction of tumor cells positive for proliferation marker Ki-67 (Tables 2 and 3).

**Table 2:** Serum enterolactone glucuronide and enterolactone sulfate levels in relation to clinical and pathological characteristics of tumors.

	Enterolactone glucuronide (nM), median(IQR)	*P* value	Enterolactone sulfate (nM), median (IQR)	*P* value
**Age**				
< 65 years (n = 43)	2.61 (0.80–7.18)	0.217	0.119 (0.063–0.249)	0.376
≥ 65 years (n = 72)	4.14 (1.10–7.37)		0.170 (0.054–0.343)	
**Gender**				
Male (n = 57)	3.64 (1.30–11.61)	0.074	0.170 (0.072–0.392)	0.163
Female (n = 58)	3.10 (0.67–6.54)		0.132 (0.042–0.230)	
**BMI**				
<25 (n = 47)	4.93 (1.34–9.33)	0.285	0.151 (0.051–0.299)	0.439
25–30 (n = 44)	2.36 (0.65–7.08)		0.121 (0.055–0.227)	
>30 (n = 22)	3.10 (1.15–6.43)		0.186 (0.063–0.412)	
**Time of operation**				
Winter (Dec–Feb) (n = 20)	3.40 (0.51–6.44)	0.066	0.148 (0.051–0.190)	0.068
Spring (Mar–May) (n = 34)	3.23 (0.93–7.07)		0.145 (0.057–0.310)	
Summer (Jun–Aug) (n=40)	6.13 (2.01–11.19)		0.213 (0.107–0.438)	
Autumn (Sep–Nov) (n=21)	1.93 (0.58–3.47)		0.081 (0.021–0.217)	
**Tumor location**				
Proximal colon (n = 49)	2.36 (0.54–6.93)	0.049	0.106 (0.025–0.229)	0.012
Distal colon (n = 28)	3.09 (0.81–6.38)		0.150 (0.067–0.250)	
Rectum (n = 38)	5.39 (1.94–11.29)		0.190 (0.105–0.443)	
**TNM Stage**				
Stage I (n = 17)	3.06 (0.62–10.32)	0.846	0.167 (0.038–0.287)	0.575
Stage II (n = 46)	3.40 (0.98–7.74)		0.147 (0.074–0.324)	
Stage III (n = 30)	3.10 (1.45–9.73)		0.170 (0.077–0.279)	
Stage IV (n = 21)	4.36 (0.90–5.57)		0.115 (0.032–0.243)	
**Depth of invasion**				
T1 (n = 4)	0.64 (0.13–9.39)	0.515	0.035 (0.007–0.197)	0.165
T2 (n = 18)	4.77 (1.05–10.76)		0.179 (0.127–0.299)	
T3 (n = 83)	3.39 (1.08–6.96)		0.145 (0.063–0.299)	
T4 (n = 9)	3.65 (1.15–8.41)		0.090 (0.020–0.308)	
**Nodal metastasis**				
N0 (n = 67)	3.14 (0.76–7.87)	0.739	0.148 (0.051–0.254)	0.296
N1 (n = 27)	5.61 (1.51–6.58)		0.183 (0.108–0.301)	
N2 (n = 19)	3.52 (0.80–9.33)		0.106 (0.022–0.249)	
**Distant metastasis**				
M0 (n = 94)	3.39 (1.02–8.93)	0.582	0.157 (0.063–0.286)	0.271
M1 (n = 21)	4.36 (0.90–6.57)		0.115 (0.032–0.243)	
**WHO Grade 1**–**3**				
Grade 1 (n = 14)	3.61 (0.48–12.98)	0.097	0.121 (0.039–0.323)	0.023
Grade 2 (n = 86)	4.31 (1.50–7.45)		0.170 (0.089–0.300)	
Grade 3 (n = 14)	1.51 (0.50–3.68)		0.053 (0.012–0.192)	
**WHO Grade 1-2/3**				
Grade 1-2 (n = 100)	3.95 (1.10–8.00)	0.032	0.169 (0.081–0.292)	0.011
Grade 3 (n = 14)	1.51 (0.50–3.68)		0.053 (0.012–0.192)	
**Tumor necrosis**				
9% or less (n = 57)	3.14 (0.72–7.35)	0.557	0.170 (0.063–0.343)	0.442
10% or more (n = 57)	3.65 (1.19–7.83)		0.132 (0.065–0.252)	
**Modified Glasgow Prognostic score (mGPS)**				
0 (n = 89)	3.77 (1.04–8.93)	0.115	0.157 (0.081–0.300)	0.088
1-2 (n = 26)	2.33 (0.78–6.18)		0.077 (0.012–0.230)	
**Diabetes**				
No (n = 90)	2.94 (0.74–6.62)	0.028	0.132 (0.046–0.247)	0.021
Yes (n = 25)	6.45 (1.97–13.83)		0.231 (0.094–0.553)	
**Antihypertensive medication**				
No (n = 52)	3.65 (1.16–7.88)	0.835	0.150 (0.065–0.238)	0.617
Yes (n = 63)	3.14 (0.98–7.18)		0.145 (0.051–0.384)	
**Cholesterol-lowering medication**				
No (n = 75)	2.73 (0.69–6.53)	0.097	0.130 (0.051–0.246)	0.094
Yes (n = 40)	4.66 (1.07–10.49)		0.190 (0.081–0.417)	

Abbreviations: IQR: interquartile range; BMI: body mass index. P values are for Mann-Whitney or Kruskal-Wallis test.

**Table 3:** Correlations between serum enterolactone levels and systemic inflammatory markers CRP and IL6, tumor cell proliferation marker Ki-67, and kidney function marker creatinine.

	^a^mg/L, ^b^pg/mL, ^c^%, ^d^μmol/L, median (IQR)	Enterolactone glucuronide		Enterolactone sulfate	
		Pearson r	*P* value	Pearson r	*P* value
Serum C-reactive protein	2.22 (0.81–8.17)^a^	−0.044	0.654	−0.081	0.412
Serum IL-6	4.92 (3.45–9.51)^b^	−0.146	0.135	−0.171	0.084
Ki-67^+^ tumor cell percentage	27.0 (13.0–53.3)^c^	0.177	0.068	0.015	0.880
Serum creatinine	59.0 (52.7–71.1)^d^	0.200	0.042	0.177	0.077

^Δ^Numbers indicate Pearson correlation coefficients (r) for logarithmically transformed variables.

### Serum enterolactone concentrations in CRC patients with concomitant diseases

We further assessed the effect of the concomitant diseases on serum enterolactone levels in CRC patients (Table 2). Hypertension- or cholesterol-lowering medication was not associated with altered serum enterolactone levels. Interestingly, patients with diabetes had higher enterolactone glucuronide and sulfate concentrations than the patients without diabetes (Glucuronide: P = 0.028 and sulfate: P = 0.021, Table 2). Previously, increased serum enterolactone concentrations have been shown in patients with diabetic renal disease^18^. Therefore, we assessed the relationship between serum levels of enterolactone and kidney function marker creatinine among CRC patients. We found a significant positive correlation between enterolactone glucuronide and creatinine (r = 0.200, P = 0.042, Table 3) and a tendency towards positive correlation between enterolactone sulfate and creatinine (r = 0.177, P = 0.077, Table 3).

### Serum enterolactone concentrations in relation to systemic inflammation markers and tumor cell proliferation

The concentrations of several circulating biomarkers and tumor prognostic parameters have been reported to be related to the systemic inflammatory response in CRC patients^19–24^. Therefore, we assessed, whether serum enterolactone levels were associated with systemic inflammatory markers. We measured serum concentrations of 13 cytokines (IL-1ra, IL-4, IL-6, IL-7, IL-8, IL-9, IL-12, IFNY, CXL10, CCL2, CCL4, CCL11 and PDGF-BB) and C-reactive protein. However, we did not find any statistically significant associations between serum enterolactone and these inflammatory markers (Table 3; some data not shown).

### Survival analysis

**Figure 1:**
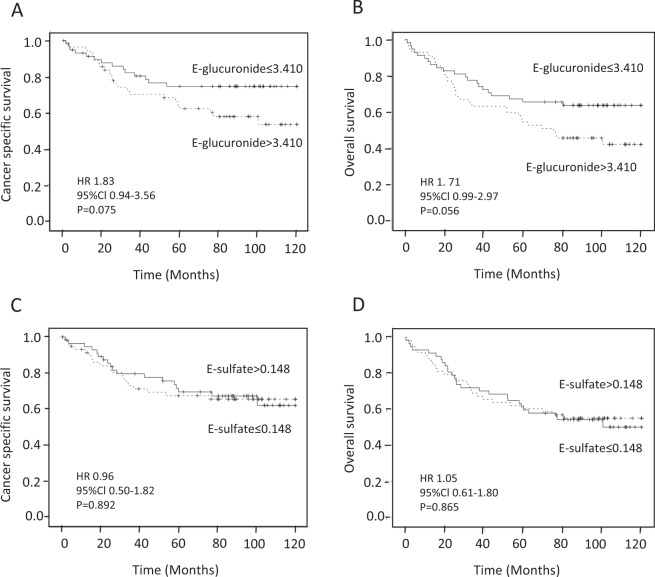
Kaplan-Meier curves showing the relationships between enterolactone glucuronides (**A**,**B**) and sulfate (**C**,**D**) in cancer specific survival (A and C, and overall survival (**B**,**D**).

We performed a 120-month survival analysis in CRC patients (Fig. 1). The receiver operating characteristic (ROC) analysis indicated that serum enterolactone levels did not significantly discriminate survivors from non-survivors (glucuronide: cancer specific survival (CSS), area under the curve: 0.552, 95% confidence interval (CI): 0.443–0.661; sulfate: CSS, area under the curve: 0.500, 95% CI: 0.390–0.610). Univariate analysis utilizing medians as cut-off points showed that serum enterolactone levels had no statistically significant association with CSS (glucuronide: hazard ratio (HR): 1.83, 95% CI: 0.942–3.561, P = 0.075 and sulfate: HR: 0.96, 95% CI 0.502–1.823, P = 0.892) or overall survival (glucuronide: HR: 1.71, 95% CI: 0.986–2.968, P = 0.056 and sulfate: HR: 1.05, 95% CI: 0.611–1.796, P = 0.865). In multivariate survival models, enterolactone glucuronide or enterolactone sulfate levels had no statistically significant association with CSS or overall survival (OS) (Table 4).

**Table 4:** Multivariate analysis of 120-month cancer-specific survival (CSS) and overall survival (OS) of CRC patients.

	CSS			OS		
	HR	95%CI	*P* value	HR	95%CI	*P* value
Age (<65 vs. ≥65)	2.47	1.12–5.47	**0**.**025**	2.35	1.22–4.54	**0**.**011**
Tumor invasion (T1–T2 vs. T3–T4)	0.68	0.21–2.16	0.510	0.90	0.38–2.12	0.801
Nodal metastases (N0 vs. N1–N2)	6.56	2.65–16.25	**4**.**7E-5**	2.52	1.35–4.68	**0**.**004**
Distant metastases (M0 vs. M1)	7.79	3.15–19.25	**9**.**0E-6**	4.73	2.31–9.67	**2**.**1E-5**
Serum enterolactone glucuronide (≤3.410 nM vs. >3.410 nM)	1.18	0.44–3.18	0.750	1.50	0.69–3.26	0.303
Serum enterolactone sulfate (≤0.148 nM vs. >0.148 nM)	0.71	0.30–1.69	0.439	0.75	0.37–1.55	0.443

Abbreviations: CI: confidence interval; HR: hazard ratio.

## Discussion

Dietary lignan derived enterolactone has been reported to possess anti-cancer activities and to be associated with lowered risk of prostate cancer and breast cancer. Our main finding is that colon but not rectal cancer patients have lower serum enterolactone concentrations than healthy controls. In CRC patients, low tumor grade, increased serum creatinine levels indicating impaired renal function, and concomitant diabetes were significantly associated with higher serum enterolactone concentrations. Enterolactone is metabolized from diet-derived lignans in a multistep process catalyzed by intestinal bacteria. For example, secoisolariciresinol diglucoside, which is a lignan found in high concentration in flaxseed, is converted to enterolactone in four sequential reactions catalyzed by phylogenetically and functionally distantly related anaerobic bacteria^25^. Accordingly, high urinary excretion of enterolactone has been associated with a high diversity of the gut microbial community^7^. In addition, low serum enterolactone levels have been associated with low fecal total bacteria and *Lactobacillus-Enterococcus* counts^26^. Thus, we hypothesize that low serum enterolactone concentrations in colon cancer patients may reflect paucity of lignan-converting bacteria in these patients, or an intracolonic environment sub-optimal to bacterial metabolism and lignan conversion. Accordingly, CRC have been linked to gut microbiota dysbiosis^27^ and antibiotics are known to reduce circulating enterolactone levels^28,29^. Unfortunately, we do not have data on the frequency of antibiotics usage in our cohort subjects.

Earlier studies have reported that enterolactone exerts anti-proliferative activities on prostate and breast cancer *in vivo*^30,31^. However, in our CRC patient cohort, serum enterolactone abundance was not associated with tumor cell proliferation, as assessed by Ki-67 immunohistochemistry (Table 3). We further hypothesized that serum enterolactone could be altered by the presence of a systemic inflammatory response to the tumor. However, we detected no association with different cytokines or CRP. Furthermore, serum enterolactone levels were not associated with tumor stage. Currently, the prognostic classification of CRC is mainly based on tumor stage, while additional prognostic parameters could help to target the tumor with more individualized treatments^32,33^. Given the reported anti-tumor effects of enterolactone, we hypothesized that high serum enterolactone could be associated with improved patient outcome. Our cohort had a 10-year survival follow-up but no significant associations were detected between serum enterolactone levels and patient survival.

We measured serum enterolactone as glucuronide and sulfate conjugates. Consistent with earlier studies in healthy subjects^34,35^ we found that glucuronide conjugate was the major circulating enterolactone metabolite in both CRC patients and controls. Enterolactone, as well as other polyphenols, are readily conjugated in the intestinal wall and in the liver^36^. Glucuronidation and sulfation are detoxification mechanisms to eliminate potentially harmful effects of enterolactone^37,38^. Tumor targeted glucuronic acid cleavage from the enterolactone-glucuronide in the tumor tissue is currently under investigation as a potential adjuvant therapy in prostate cancer^39^.

We found that CRC patients with diabetes had increased serum enterolactone levels. A previous study found elevated serum enterolactone levels in patients with diabetic nephropathy and a strong correlation between serum enterolactone and creatinine levels^18^. Accordingly, our results support the positive correlation between serum enterolactone glucuronide and creatine, suggesting that decreased urinary secretion of enterolactone may contribute to the increased serum enterolactone levels in a subset of CRC patients.

Earlier reported determinants of serum enterolactone concentrations include consumption of lignan-containing foods, constipation, smoking and BMI^34,40^. Especially, lignan-containing plant foods such as grains, fruits and vegetables are enriched with the dietary fiber, phytoestrogen, and unsaturated fatty acids. Increased dietary intake of lignans or fortification of food with the plant lignans have resulted in increased serum enterolactone levels^2,3,41^. In the Nordic population, a healthy diet includes apples and berries, roots and cabbage, rye, oats, barley, low-fat milk products, rapeseed oil, and fish^42,43^. However, in our CRC cohort detailed food intake questionnaires were not available. Nevertheless, we did not detect statistically significant association between serum enterolactone levels and the BMI of CRC patients.

Our CRC cohort was cross sectional study with a long follow-up period from a single province in Northern Finland (65° latitude). The study was limited by its sample size, comprising 115 CRC patients and 76 healthy controls. Due to cross-sectional study design, we were not able to assess, whether enterolactone reduced the risk for colon cancer^15^ and/or affected colon cancer progression at early stages. Therefore, additional studies are required to further clarify the significance of the decreased serum enterolactone levels in colon cancer patients.

In conclusion, we found significantly decreased levels of enterolactone in colon cancer, but not in rectal cancer patients. Since a diverse gut microbiota plays a crucial role for an efficient conversion of plant to enterolignans, microbiome homeostasis may be disturbed in patients with colon, but not with rectal cancer, which requires further investigation. Serum enterolactone levels were not significantly associated with patient gender or age, tumor stage, systemic inflammatory markers, and survival.

## Methods

### Patients and controls

Preoperative blood samples were collected from 149 CRC patients, operated in the Oulu University Hospital between April 2006 and January 2010^44^. Patients with earlier or simultaneously diagnosed other malignant diseases were excluded. Thirty-two of the 149 (21.5%) patients, who had received preoperative radiotherapy or chemo radiotherapy (RT/CRT), were excluded from the analyses due to the possible confounding effects to the local tumor characteristics^45^. Four of the 149 (3.4%) cases were not applicable to this study due to insufficient sample material. Clinical data was collected from the clinical records and a questionnaire. The 10 year follow-up data was acquired from the clinical records and Statistics Finland^46,47^. Age and sex matched control serum samples were acquired from healthy voluntary blood donors (Finnish Red Cross, Oulu, Finland; n = 36, age < 65 years) and cataract surgery patients (Oulu University Hospital; n = 50, age ≥ 65 years).

The study was accepted by the Ethical Committee of the Oulu University Hospital (42/2005, 122/2009) and performed according to the National Guidelines and the principles of the Declaration of Helsinki. All the patients and controls signed an informed consent to participate in the study.

### Histopathological analysis

The tumors were staged according to TNM6^48^ and graded according to the WHO 2010 criteria^49^. The percentage of tumor tissue showing coagulative necrosis was evaluated by inspecting all the available hematoxylin and eosin stained tumor sections^50^. Tumor necrosis was specified as an area with increased eosinophilia and nuclear shrinkage, fragmentation and disappearance^50^. The proliferation index, denoting the percentage of Ki-67 positive tumor cells in the hotspots, was determined as described earlier^50^.

### Analysis of blood samples

Preoperative serum samples were collected in tubes without clot activator. The samples were centrifuged and stored at −70 °C until the analysis. Serum inflammatory markers were analyzed with Bio-Plex Pro Human pre-manufactured 27-Plex Cytokine Panel (Bio-Rad, Hercules, CA, USA)^44^. A total of 13 of 27 (48.1%) cytokines (IL-1ra, IL-4, IL-6, IL-7, IL-8, IL-9, IL-12, IFNY, CXL10, CCL2, CCL4, CCL11 and PDGF-BB) had three or fewer values outside the assay working range and were included in the study^44^. Serum CRP levels and serum albumin levels were measured in the laboratory of Oulu University Hospital and mGPS was calculated from serum CRP and albumin values^44,51^. Serum creatinine levels were analyzed with nuclear magnetic resonance metabolomics platform, equipped with Bruker AVANCE III 500 MHz and Bruker AVANCE III 600 MHz spectrometers (Bruker, Billerica, MA, USA)^19,52^.

### Enterolactone measurements

Serum enterolactone concentrations were quantified using a novel high-throughput liquid chromatography–mass spectrometry (LC-MS)/MS method by measuring the free enterolactone or intact form of either glucuronide or sulphate conjugated enterolactone, as previously described^34^. The method was validated according to the guidelines of the U.S. Food and Drug Administration (FDA) and the European Medicines Agency. Internal standards and their stability was tested previously^53^. Intrabatch accuracy, precision and recovery of enterolactone were tested at low, medium and high concentrations and the relative standard deviation did not exceed 15%. Briefly, standard curves and test serum sample were prepared using standards of (±)-enterolactone-mono-β-D-glucuronide and (±)-enterolactone monosulfate ammonium salt from ReseaChem (Burgdorf, Switzerland) and enterolactone from Plantech (Berkshire, UK). Serum samples were cleaned in solid phase extraction (SPE) hydrophilic/lipophilic balanced (HBL) 96-well plates from Waters (Torrance, CA, USA) and eluted with 50/40/10% acetonitrile (ANC)/methanol (MeOH)/H_2_O and internal standard (Glycine-1 13C) was added to the eluate. The LC-MS/MS measurements were performed on a microLC 200 series from Eksigent/AB Sciex (Redwood City, CA, USA) and QTrap 500 mass spectrometer from AB Sciex equipped with an ESI source. The microLC was equipped with a phenyl column from Eksigent/AB Sciex (Redwood City, CA, USA). The data were analysed using the Analyst software 1.6 from AB Sciex (Framingham, MA, USA).

### Statistical analyses

Statistical analyses were performed using IBM SPSS Statistics for Windows version 22.0 (IBM Corporation, Armonk, NY, USA). Normally distributed continuous variables are presented as mean (standard deviation, SD), whereas other continuous variables are presented as median (interquartile range, IQR). Correlations between two continuous variables were presented as Pearson correlation coefficients (r). Statistical significances of the differences in serum enterolactone levels between the different study groups and categorical variables were analyzed by Mann-Whitney *U* test or Kruskal-Wallis test. ROC analysis was used to evaluate the capacity of the serum enterolactone level to discriminate survivors from non-survivors. Cox regression models were used in the survival analyses. In all the tests P < 0.05 was considered statistically significant.

## Data Availability

All data generated or analyzed during this study are available from the corresponding author on reasonable request.
